# *Caenorhabditis elegans *behavioral genetics: where are the knobs?

**DOI:** 10.1186/1741-7007-8-69

**Published:** 2010-06-08

**Authors:** Leon Avery

**Affiliations:** 1 Department of Molecular Biology, University of Texas Southwestern Medical Center, 6000 Harry Hines Blvd, Dallas, TX 75390-9148, USA

## Abstract

Thousands of behavioral mutants of *Caenorhabditis elegans *have been studied. I suggest a set of criteria by which some genes important in the evolution of behavior might be recognized, and identify neuropeptide signaling pathways as candidates.

## Commentary

Evolution occurs by the accumulation of genetic changes. Behavior is under genetic control and evolves in response to selection. It is thought (at least this seems to be the view of prominent textbooks [1,2]) that behavior evolves in large part by changes in quantitative characteristics such as the intensity or frequency of a behavior, the thresholds for eliciting a particular response, or the relative timing of component actions. This implies the existence of genes whose sequences control the values of numbers that determine behavior. I like to think of an animal as a device with a complicated control panel - the genome - covered with buttons and switches and knobs - the genes. Mutation and natural selection turn the knobs to adjust behavior so as to optimize fitness in the environment in which the animal finds itself.

For almost 50 years geneticists studying the nematode worm *Caenorhabditis elegans *have been isolating and studying behavioral mutants. (See Box 2 for an explanation of what I mean by 'behavior'.) Does this work tell us anything about how behavior evolves? In particular, can it identify candidates for the knobs - genes whose sequences move the numbers that control behavior?

**Box 2:**
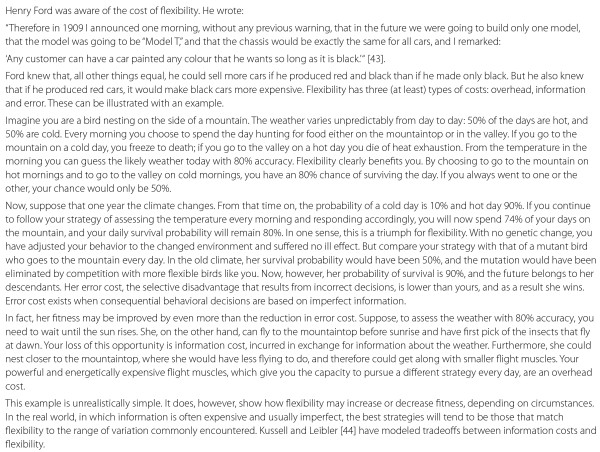
What is behavior?

## A brief overview of *C. elegans *behavioral genetics

Behavioral genes have figured prominently in *C. elegans *genetics since Sydney Brenner began isolating mutants over 40 years ago. Of the 95 genes listed in Table 4 of Brenner's first paper on the genetics of *C. elegans*, 57 affect nervous system function and behavior. (The others affect morphology (29) or muscle contraction (9); another 5 of Brenner's 100 genes are no longer thought to be distinct genes.) In part this is because Brenner and his postdocs and students were interested in the function of the nervous system, but it was also a consequence of technical constraints. Worms are morphologically simple. Unlike mice or flies, for instance, which bristle with external spikes, hairs and protuberances in a variety of shapes and colors, there is not much to see on the outside of a worm. The insides are visible and a little more complex, but most of the obvious features are too important to mess with in any serious way.

In contrast, viable and visible behavioral mutants are easy to isolate and work with. Many behavioral abnormalities are obvious under dissecting microscope observation. And viable mutants are common because under laboratory conditions, hermaphrodites can survive and reproduce even with a largely nonfunctional nervous system. We now have at least some information about the functions of most of the 118 types of neurons in the hermaphrodite [3,4], and only one of them, CAN, is known to be essential in the lab. (Worms lacking CAN wither and fail to grow, but its exact function is still not clear.) The feeding motor neuron M4 was reported to be essential [5], but it has since been found that M4-minus worms are viable and fertile when grown on small bacteria (JT Chiang, M Steciuk, B Shtonda, and L Avery, unpublished). Feeding is essential, but the motion of the feeding muscles continues in a slow, uncoordinated, but still functional way in the absence of the motor neurons that control them [6]. Since they self-fertilize, hermaphrodites do not need to mate to reproduce. We grow them literally swimming in food, so they do not need to move to find it. Egg-laying is not essential for fertility, as unlaid eggs hatch inside the mother, eat her, and escape [7,8]. Defects in mechanosensation [9], thermotaxis [10], chemosensation [11] and many other behaviors that are probably important or essential in the wild have little effect on survival in the lab.

Consequently, mutations that drastically reduce or eliminate the function of most of the nervous system are easily isolated. This has been a great advantage for the investigation of fundamental neuronal processes such as synaptic transmission [12]. Many of the genes identified by Brenner affect such processes as neurotransmitter synthesis, vesicle loading, active zone formation, vesicle fusion, or postsynaptic response [12]. Similar mutations in other animals are almost always lethal.

In most cases, one need only look at one of these mutants to be convinced that the mutation is unlikely to be important in evolution. *unc-18*, for instance, is essential for synaptic transmission [13], and *unc-18 *mutants are almost totally paralyzed, feed slowly, and grow and reproduce much more slowly than wild type [14]. Very few of the behavioral mutations studied in *C. elegans *labs are convincing candidates for a useful setting on the evolutionary control panel. The reason for this is selection bias. Mutations that cause large, obvious changes are the easiest to identify and study. And as geneticists we focus on null or strong loss-of-function mutations, as these typically provide the most easily interpretable information about the function of the wild-type gene.

However, although the *mutations *we study are unlikely fodder for evolution, the *genes *they identify might, if their functions were more subtly altered, tweak behavior in adaptive ways.

## Why evolution is hard to do

The behavior of an animal is a complicated machine with many interlocking gears. If you change one part, you must change the connecting parts, too, if the machine is not to break. Consider the changes that would be necessary to adapt an animal that evolved under conditions of stable food supply to a new environment in which food supply is unpredictable. If the food supply is stable, it is wasteful to store lots of fat - the energy is better devoted to attaining reproductive age as rapidly as possible and producing progeny. If food is unpredictable, it makes sense to eat more than you need and stock away some of the surplus as fat, so that you can survive lean times. But without other adaptations, simply increasing the amount that you eat would serve little purpose. Without increased expression of digestive enzymes, an increase in feeding rate might have little effect. Physiological changes such as slowing reproduction and increasing the expression of anabolic enzymes in storage organs would be necessary to allow the accumulation of reserves. Foraging strategies would need to change in order to match the accumulation of reserves to the risk of hunger. And, of course, reserves are only valuable if they are used when needed. You need to gather information about nutritional stress, or the possibility of stress, and adjust behavior and physiology in response.

**Figure 1:**
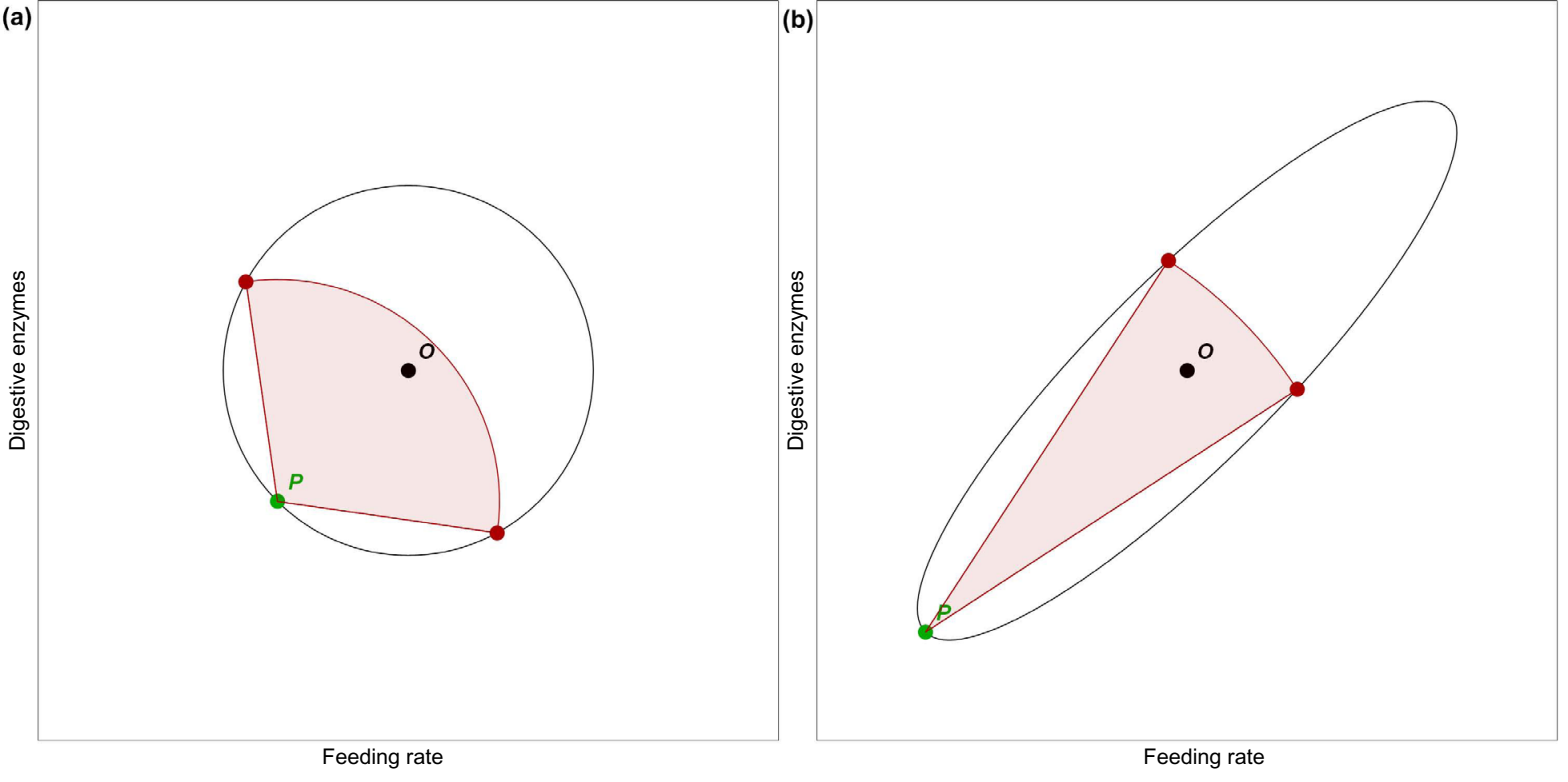
**Adaptation can be represented by motion in phenotype space**. This figure shows a hypothetical phenotype space for feeding rate and expression of digestive enzymes. **(a) **Point *O *is the optimal phenotype, and *P *is the animal's current phenotype. Now suppose that a mutation changes the phenotype. This can be thought of as a step from *P *to some other point. If the new point is closer to *O *than *P *was, that is, if it is within the black circle, then it is adaptive. For very small steps the probability of an adaptive step is close to 50%. The set of adaptive points for a step 1.2 times the distance from *P *to *O *is shown by the red arc; the probability of improved adaptation is 30%. This probability continues to decrease as step size increases, until it becomes 0 for steps of size 2 or greater. **(b) **Part (a) assumes no interaction between feeding rate and digestive enzyme expression. In reality, correlated changes are more likely to be adaptive than changes in which one increases while the other decreases. This can be represented by changing the target area to an ellipse. It is still the case that small changes are more likely to be adaptive than big changes. However, much larger adaptive changes are now possible, if they are along the axis of the ellipse. The figure shows a change of size 2.4, which would always be maladaptive for a circular target of equal area. Here the probability of improvement is 7%.

Of course, this is not a new observation, nor is the problem unique to behavior. Every extant living thing is adapted to its environment through the action of many complicated machines whose parts must work together, and which are therefore difficult to change. In *The Genetical Theory of Natural Selection *[15], Ronald Fisher offered a geometric analogy to understand the problem of adaptation. It begins by thinking of phenotype as a point in space. Each dimension of the space represents some aspect of the phenotype that can vary. For instance, one might plot average feeding rate along the *x*-axis and digestive enzyme expression on the *y*-axis (Figure 1a). The optimal combination of these two (which will depend on the environment) is a point *O *in this phenotype space; the actual phenotype another point, *P*. Fisher suggested that the degree of adaptation might be represented by the distance from *O *to *P*. The target of adaptation, comprising all phenotypes better than *P*, is located in a disk centered at *O *whose edge passes through *P*.

Now, suppose a mutation causes a change in the phenotype: what is the probability that the change will be an improvement? For very small changes, the chance that a random change moves *P *towards *O *is about equal to the chance that it moves away. Thus, the probability of hitting within the target and improving adaptation is 50%. But this probability decreases progressively as the size of the change increases (see demonstration in Additional file 1). If we were considering three changes - for example, eat more, express digestive enzymes, and slow reproduction - the target would be a ball centered at *O*, and random changes would be even less likely to be inside. Most likely, real adaptations occur in a phenotype space of far more than three dimensions. Fisher showed that when there are many dimensions along which adaptation occurs, only very small changes are likely to improve adaptation. This is consistent with the intuitive perception that changes to a complex machine with many parts are far more likely to damage it than improve it. Fisher therefore argued that evolution would occur only by small steps.

The example suggests, however, that this picture is too simple. One adaptation does not affect fitness independently of others - rather, they interact. Increased expression of digestive enzymes will have only a limited effect if the rate of feeding does not change. An increased feeding rate may have little effect unless digestive enzyme expression increases. But simultaneous increases in these two quantities may result in a far greater change in nutrient intake than the sum of the two individual changes. One can easily imagine that in an appropriate environment, the two changes together might improve fitness.

Thus, some directions in phenotype space make more functional sense than others. Increasing enzyme expression while decreasing feeding (movement toward the upper left in Figure 1) is almost certainly a bad idea, no matter where you are, but increasing them together (movement toward the upper right) may well improve fitness. The target for improved adaptation, rather than being a circle or a ball, is more like an ellipsoid (Figure 1b). Although random changes are still unlikely to be improvements, changes along the length of the ellipsoid are far more likely to hit within it than changes in other directions. To the extent that we understand the function of the behaviors, we can recognize these directions as coherent changes in many behaviors that together serve a common purpose.

If an animal is poorly adapted to its environment, a likely cause is that the environment has recently changed. Changes in the environment correspond to motion of the point *O*. Changes in the environment are not random. For instance, a sustained increase in food availability is more likely to be accompanied by sustained increases in population density and predator activity than by decreases. This means that *O *is more likely to move in some directions than in others. If an animal is maladapted because of a recent change in its environment, a mutation to adapt an animal to the new environment should move *P *in the same direction that *O *moved.

These arguments suggest that, if they exist, genes that satisfy the following criteria might be particularly important in behavioral evolution. First, the gene affects multiple related behaviors (pleiotropy). Second, the behaviors are affected in such a way that together they make functional sense (functional coherence). Third, these combined behavioral changes are in a direction that is an appropriate response to a likely environmental change (environmental responsiveness).

## Signals with significance

Can we identify genes that meet these criteria? They seem extraordinarily demanding. It is not just that they constrain the genes of interest. Our ability to recognize them is also a problem. To evaluate them, we require at least a crude understanding of how the environment is likely to change, and what sort of behavioral changes represent a functionally coherent response. Are there any genes that match?

In fact, although the majority of *C. elegans *genes studied do not fit these criteria, some do. Examples are *egl-4*, which encodes cGMP-dependent protein kinase (PKG), and *daf-2*, which encodes the insulin/insulin-like growth factor (IGF) receptor. *egl-4 *affects a variety of food-seeking behaviors. Normal worms alternate between two modes of locomotion: roaming, in which they search for good food; and dwelling, when they consume what they have found [16,17]. Loss of *egl-4 *function causes worms to roam, as if continually searching for better food [16]. Wild-type worms, given abundant high-quality food, will eventually stop eating and become quiescent; *egl-4 *loss-of-function worms continue to eat, while *egl-4 *gain-of-function mutants become quiescent even in poor food [18]. *egl-4 *loss-of-function mutants grow bigger than wild type [16,19], while the gain-of-function mutant is small [20]. These phenotypes can be understood as responses to a change in the reliability and quality of the food supply: specifically, *egl-4 *function makes worms act in a way that is appropriate for a reliable, high-quality food supply. *egl-4 *also affects egg-laying [20,21] and chemosensory adaptation [22]. The *Drosophila *PKG gene *foraging *has similar effects on fly behavior [23,24]. *foraging *is polymorphic in wild populations and affects fitness in laboratory selection experiments. The expression of its homolog in honeybees correlates with the transition from nurse to forager caste [25].

*daf-2 *activity is controlled by nutritional state, and regulates the balance between growth and reproduction on the one hand, versus survival and safety on the other. *daf-2 *loss-of-function mutants were initially identified because they become dauer larvae even under favorable growth conditions [26]. The dauer larva is a developmental diapause normally entered by wild-type worms only under unfavorable conditions. Dauers can survive harsh conditions for many times the normal lifespan without aging. But *daf-2 *has since been shown to profoundly affect physiology. *daf-2 *loss-of-function mutants are bullet-proof: they have up to twice the lifespan of wild-type worms [27] and are resistant to pathogens [28] and a wide variety of stresses (see, for example [29,30]). They grow more slowly than wild-type worms (Y You, A Artyukhin, unpublished observations) and synthesize and store more fat than wild-type [31].

There are other genes that meet the criteria of pleiotropy, functional coherence, and environmental responsiveness. Mutations in other genes in the insulin signaling pathway, not surprisingly, have phenotypes either similar or opposite to *daf-2 *loss-of-function. Other examples would be genes in the *daf-7 *transforming growth factor beta (TGF-β) and *flp-18 *neuropeptide signaling pathways. The neuropeptide receptor gene *npr-1*, which affects social foraging in response to atmospheric gases [32-36], is also a candidate, although in this case it is less obvious what sort of environmental change, if any, its activity might represent an appropriate response to.

This short list of genes is subjective and likely to be incomplete, but still it is intriguingly nonrandom. First, all these genes are concerned with responses to food or nutrition. This may not be very informative: almost every known *C. elegans *behavior is influenced by food, and changes in the quantity, quality and reliability of the food supply are among the most easily recognized environmental variables. Second, and more interesting, all the genes affect specific peptide hormone signaling pathways. (*egl-4*, for instance, is thought to affect the signaling pathways of two TGF-βs: DBL-1 [37] and DAF-7 [18]).

At some cost in precision, the statement that a signal meets the criteria of pleiotropy, functional coherence and environmental responsiveness can be summarized as a claim that it has significance. The signal carries information about some important characteristic of the environment and provokes appropriate responses. Put this way, it is not, after all, so surprising that there are such genes. Behavior exists to allow an animal to adapt to changes in its environment. Of course animals have signals that signify changes in the environment and provoke appropriate responses. Within the life of an animal the pathways are regulated by all the mechanisms by which the function of a gene product may be regulated: gene expression, post-translational modification, subcellular location, and so on. But over generations they may also be regulated by changes in the genes that encode them.

The evolution of behavior may be different in this respect from that of other biological processes such as development. Much of development happens once and is finished, and cannot then respond to changes in the environment within the lifetime of an animal. This is of course an oversimplification, but it is fair to say that, on time scales short compared to the life cycle, behavior accounts for more of an animal's flexibility in responding to the environment than does development. Consequently, there is less need for developmental signals to signify a changing environment. Indeed, although signaling is important in development, most developmental signals can be understood not as conveying information about the animal's environment, but rather the local environment of a cell within the animal.

The idea that evolution of behavior occurs by changes in signaling pathways raises a question. I have implied that a mutation that increased the activity of the DAF-2 insulin/IGF receptor might make the worm more fit for environments in which food is abundant. But the activity of DAF-2 is regulated in real time by food abundance. Doesn't this behavioral flexibility trump genetic adaptation? Wouldn't the fittest organism be one that can adapt to any environment it will encounter?

The answer is sometimes yes, sometimes no. Flexibility has costs (Box 3). The Worm for All Seasons, capable of responding to every environmental change that it is likely to encounter during its long-term evolution, will not necessarily be fitter than a less flexible worm able to respond behaviorally only to those changes that occur frequently in its environment.

**Box 3:**
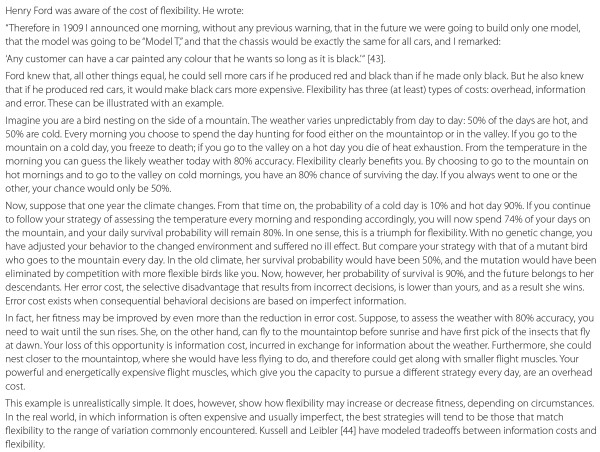
The cost of flexibility

## Is it true?

Are these genes really the volume knobs that are turned by the evolution of behavior? To my knowledge, this has not yet been tested in *C. elegans*. (However, the idea is consistent with work suggesting the importance of oxytocin/vasopressin signaling in the regulation and evolution of monogamy in voles [38] and possibly even in humans [39].) Unfortunately, although genes such as *daf-2 *and *egl-4 *may be particularly important in the evolution of behavior, it is unlikely that the mutations that have been studied in the lab are. Most of these mutations were found in screens biased towards large, obvious effects. In most cases the mutant worms are obviously crippled by changes in gene activity that are far too large (null mutations, for instance) and would not be competitive in the wild.

The hypothesis could be tested, however. First, laboratory evolution experiments or recombinant inbred lines could be used to identify genes that confer an advantage under selection for changed behavior. It is particularly easy to make hermaphrodite recombinant inbred lines [40], and some behavioral studies have already been done [41]. Second, one could look at variation in natural populations. One might expect to see more than average polymorphism in genes of these signaling pathways, accompanied by signals of stronger selection. Third, one could compare the genes from other *Caenorhabditis *species, several of which have now been sequenced. Even without knowing how the niches of these species differ, it is likely that optimal behavior differs, and therefore that there would be a higher frequency of functional polymorphisms in these genes than in others.

## Supplementary Material

Additional file 1 **Demonstration**. This additional file contains the demonstration pspace_v2.nbp, which allows these relationships to be explored interactively. This requires Mathematica Player, which can be downloaded from [42]. Click here for file

## References

[B1] Futuyma DJ Evolutionary Biology 1998 Sunderland, MA: Sinauer

[B2] Alcock J Animal Behavior: An Evolutionary Approach 2009 Sunderland, MA: Sinauer

[B3] WormAtlas http://www.wormatlas.org/

[B4] White JG Southgate E Thomson JN Brenner S The structure of the nervous system of the nematode *Caenorhabditis elegans* Philos Trans R Soc Lond B Biol Sci 1986 314 1 340 10.1098/rstb.1986.0056 22462104

[B5] Avery L Horvitz HR A cell that dies during wild-type *C. elegans *development can function as a neuron in a ced-3 mutant Cell 1987 51 1071 1078 3690660 10.1016/0092-8674(87)90593-9 PMC3773210

[B6] Avery L Horvitz HR Pharyngeal pumping continues after laser killing of the pharyngeal nervous system of *C. elegans* Neuron 1989 3 473 485 2642006 10.1016/0896-6273(89)90206-7

[B7] Ferguson EL Horvitz HR Identification and characterization of 22 genes that affect the vulval cell lineages of the nematode *Caenorhabditis elegans* Genetics 1985 110 17 72 3996896 10.1093/genetics/110.1.17 PMC1202554

[B8] Trent C Tsung N Horvitz H Egg-laying defective mutants of the nematode *Caenorhabditis elegans* Genetics 1983 104 619 647 11813735 10.1093/genetics/104.4.619 PMC1202130

[B9] O'Hagan R Chalfie M Mechanosensation in *Caenorhabditis elegans* Int Rev Neurobiol 2006 69 169 203 16492465 10.1016/S0074-7742(05)69006-X

[B10] Mori I Sasakura H Kuhara A Worm thermotaxis: a model system for analyzing thermosensation and neural plasticity Curr Opin Neurobiol 2007 17 712 719 18242074 10.1016/j.conb.2007.11.010

[B11] Bargmann CI Chemosensation in *C. elegans* WormBook 2006 1 29 18050433 10.1895/wormbook.1.123.1 PMC4781564

[B12] Richmond J Synaptic function WormBook 2005 1 14 18050398 10.1895/wormbook.1.69.1 PMC4781477

[B13] Weimer RM Richmond JE Davis WS Hadwiger G Nonet ML Jorgensen EM Defects in synaptic vesicle docking in unc-18 mutants Nat Neurosci 2003 6 1023 1030 12973353 10.1038/nn1118 PMC3874415

[B14] Hosono R Hekimi S Kamiya Y Sassa T Murakami S Nishiwaki K Miwa J Taketo A Kodaira KI The *unc-18 *gene encodes a novel protein affecting the kinetics of acetylcholine metabolism in the nematode *Caenorhabditis elegans* J Neurochem 1992 58 1517 1525 1347782 10.1111/j.1471-4159.1992.tb11373.x

[B15] Fisher R The Genetical Theory of Natural Selection 1958 New York: Dover Publications in press

[B16] Fujiwara M Sengupta P McIntire SL Regulation of body size and behavioral state of *C. elegans *by sensory perception and the EGL-4 cGMP-dependent protein kinase Neuron 2002 36 1091 1102 12495624 10.1016/s0896-6273(02)01093-0

[B17] Shtonda BB Avery L Dietary choice behavior in *Caenorhabditis elegans* J Exp Biol 2006 209 89 102 16354781 10.1242/jeb.01955 PMC1352325

[B18] You YJ Kim J Raizen DM Avery L Insulin, cGMP, and TGF-beta signals regulate food intake and quiescence in *C. elegans*: a model for satiety Cell Metab 2008 7 249 257 18316030 10.1016/j.cmet.2008.01.005 PMC3786678

[B19] Hirose T Nakano Y Nagamatsu Y Misumi T Ohta H Ohshima Y Cyclic GMP-dependent protein kinase EGL-4 controls body size and lifespan in C. elegans Development 2003 130 1089 1099 12571101 10.1242/dev.00330

[B20] Raizen DM Cullison KM Pack AI Sundaram MV A novel gain-of-function mutant of the cyclic GMP-dependent protein kinase egl-4 affects multiple physiological processes in *Caenorhabditis elegans* Genetics 2006 173 177 187 16547093 10.1534/genetics.106.057380 PMC1461420

[B21] Desai C Horvitz HR *Caenorhabditis elegans *mutants defective in the functioning of the motor neurons responsible for egg laying Genetics 1989 121 703 721 2721931 10.1093/genetics/121.4.703 PMC1203655

[B22] L'Etoile ND Coburn CM Eastham J Kistler A Gallegos G Bargmann CI The cyclic GMP-dependent protein kinase EGL-4 regulates olfactory adaptation in *C. elegans* Neuron 2002 36 1079 1089 12495623 10.1016/s0896-6273(02)01066-8

[B23] Osborne KA Robichon A Burgess E Butland S Shaw RA Coulthard A Pereira HS Greenspan RJ Sokolowski MB Natural behavior polymorphism due to a cGMP-dependent protein kinase of *Drosophila* Science 1997 277 834 836 9242616 10.1126/science.277.5327.834

[B24] Raizen DM Zimmerman JE Maycock MH Ta UD You YJ Sundaram MV Pack AI Lethargus is a *Caenorhabditis elegans *sleep-like state Nature 2008 451 569 572 18185515 10.1038/nature06535

[B25] Kaun KR Sokolowski MB cGMP-dependent protein kinase: linking foraging to energy homeostasis Genome 2009 52 1 7 19132066 10.1139/G08-090

[B26] Riddle DL A genetic pathway for dauer larva formation in *C. elegans* Stadler Genet Symp 1977 9 101 120

[B27] Kenyon C Chang J Gensch E Rudner A Tabtiang R A *C. elegans *mutant that lives twice as long as wild type Nature 1993 366 461 464 8247153 10.1038/366461a0

[B28] Evans EA Chen WC Tan MW The DAF-2 insulin-like signaling pathway independently regulates aging and immunity in *C. elegans* Aging Cell 2008 7 879 893 18782349 10.1111/j.1474-9726.2008.00435.x PMC2630471

[B29] Scott BA Avidan MS Crowder CM Regulation of hypoxic death in *C. elegans *by the insulin/IGF receptor homolog DAF-2 Science 2002 296 2388 2391 12065745 10.1126/science.1072302

[B30] Mendenhall AR LaRue B Padilla PA Glyceraldehyde-3-phosphate dehydrogenase mediates anoxia response and survival in *Caenorhabditis elegans* Genetics 2006 174 1173 1187 16980394 10.1534/genetics.106.061390 PMC1667098

[B31] Perez CL Van Gilst MR A ^13^C isotope labeling strategy reveals the influence of insulin signaling on lipogenesis in *C. elegans* Cell Metab 2008 8 266 274 18762027 10.1016/j.cmet.2008.08.007

[B32] Hallem EA Sternberg PW Acute carbon dioxide avoidance in *Caenorhabditis elegans* Proc Natl Acad Sci USA 2008 105 8038 8043 18524955 10.1073/pnas.0707469105 PMC2430355

[B33] Pocock R Hobert O Hypoxia activates a latent circuit for processing gustatory information in *C. elegans* Nat Neurosci 13 610 614 20400959 10.1038/nn.2537 PMC3733994

[B34] de Bono M Bargmann CI Natural variation in a neuropeptide Y receptor homolog modifies social behavior and food response in *C. elegans* Cell 1998 94 679 689 9741632 10.1016/s0092-8674(00)81609-8

[B35] Bretscher AJ Busch KE de Bono M A carbon dioxide avoidance behavior is integrated with responses to ambient oxygen and food in *Caenorhabditis elegans* Proc Natl Acad Sci USA 2008 105 8044 8049 18524954 10.1073/pnas.0707607105 PMC2410288

[B36] Rogers C Persson A Cheung B de Bono M Behavioral motifs and neural pathways coordinating O_2 _responses and aggregation in *C. elegans* Curr Biol 2006 16 649 659 16581509 10.1016/j.cub.2006.03.023

[B37] Nagamatsu Y Ohshima Y Mechanisms for the control of body size by a G-kinase and a downstream TGFbeta signal pathway in *Caenorhabditis elegans* Genes Cells 2004 9 39 47 14723706 10.1111/j.1356-9597.2004.00700.x

[B38] Hammock EA Gene regulation as a modulator of social preference in voles Adv Genet 2007 59 107 127 17888796 10.1016/S0065-2660(07)59004-8

[B39] Walum H Westberg L Henningsson S Neiderhiser JM Reiss D Igl W Ganiban JM Spotts EL Pedersen NL Eriksson E Lichtenstein P Genetic variation in the vasopressin receptor 1a gene (AVPR1A) associates with pair-bonding behavior in humans Proc Natl Acad Sci USA 2008 105 14153 14156 18765804 10.1073/pnas.0803081105 PMC2533683

[B40] Rockman MV Kruglyak L Recombinational landscape and population genomics of *Caenorhabditis elegans* PLoS Genet 2009 5 e1000419 19283065 10.1371/journal.pgen.1000419 PMC2652117

[B41] Doroszuk A Snoek LB Fradin E Riksen J Kammenga J A genome-wide library of CB4856/N2 introgression lines of *Caenorhabditis elegans* Nucleic Acids Res 2009 37 e110 19542186 10.1093/nar/gkp528 PMC2760803

[B42] Mathematica Player http://www.wolfram.com/products/player

[B43] Ford H Crowther S My Life and Work 1922 Garden City, NY: Doubleday, Page & Company

[B44] Kussell E Leibler S Phenotypic diversity, population growth, and information in fluctuating environments Science 2005 309 2075 2078 16123265 10.1126/science.1114383

